# Analyzing the Long Term Cohesive Effect of Sector Specific Driving Forces

**DOI:** 10.1371/journal.pone.0152487

**Published:** 2016-03-31

**Authors:** Yonatan Berman, Eshel Ben-Jacob, Xin Zhang, Yoash Shapira

**Affiliations:** 1 School of Physics and Astronomy, Tel-Aviv University, Tel-Aviv, Israel; 2 College of Transport and Communication, Shanghai Maritime University, Shanghai, China; East China University of Science and Technology, CHINA

## Abstract

Financial markets are partially composed of sectors dominated by external driving forces, such as commodity prices, infrastructure and other indices. We characterize the statistical properties of such sectors and present a novel model for the coupling of the stock prices and their dominating driving forces, inspired by mean reverting stochastic processes. Using the model we were able to explain the market sectors’ long term behavior and estimate the coupling strength between stocks in financial markets and the sector specific driving forces. Notably, the analysis was successfully applied to the shipping market, in which the Baltic dry index (BDI), an assessment of the price of transporting the major raw materials by sea, influences the shipping financial market. We also present the analysis of other sectors—the gold mining market and the food production market, for which the model was also successfully applied. The model can serve as a general tool for characterizing the coupling between external forces and affected financial variables and therefore for estimating the risk in sectors and their vulnerability to external stress.

## Introduction

As a part of the effort to study financial markets, sectors within the market, in which stocks are strongly interacting are particularly analyzed [1–10]. Due to their nature, some of these sectors are affected by external driving forces. For example, the energy sector stocks are strongly affected by the oil and natural gas prices. Such sectors are expected to differ in various aspects from the market index, due to their dependence on external forces. Analyzing the behavior of such sectors is meaningful for the overall understanding of the stock market. In particular, the importance of such sectors is relevant due to their role in diversifying the market, by incorporating information on external indices and forces [7–9, 11–14].

The aim of this paper is to characterize the statistical properties of sectors in the context of their coupling with external driving forces. The characterization is accompanied by proper modeling of this coupling, resulting in the cointegration of the stock prices and the driving forces, as well as in Granger causality between the index and the stock prices [15, 16]. Using the model, it is possible to estimate the coupling strength between the external forces and the stock prices in addition to estimating the risk in specific sectors and assessing their vulnerability to external stress.

First, we devise a theoretical model for a set of random walking stocks coupled to an external force or index. We then analyze the statistical properties of the shipping sector, as a typical example of a sector in the financial market, and compare the model results with these statistical properties. In order to further validate the model, it is applied to the gold mining and food production sectors as well. Finally, a discussion of the results and a summary of the work are presented.

## A coupling model for a market sector with an external driving force

As an initial step, we devise a theoretical model for the behavior of a set of stocks with an external driving force. The main rationale behind the model is the market mechanism, sometimes apparent in fundamental and technical analysis, in which prices of indices and various other variables are treated as indicators for future outcome. On the daily level, the change of each stock can be treated as random, added by a coupling term, which prevents the stock price from significantly diverging on the long run from the driving force. This mechanism produces an effective interaction between the stocks, creating a process of cointegration as well as Granger causality between the stock prices and the index.

Assuming the external driving force is a time series *I*_*t*_, with *t* = 1, …, *N*, we produce *M* time series for the price of *M* stocks, denoted as $X_{t}^{i}$, with *i* = 1, …, *M*. We arbitrarily set the initial price of each stock to be 1, and assume, without the loss of generality, that *I*_1_ = 1. We randomly create a set of random noise series $\omega_{t}^{i}$, which follow N(0,0.0001) and define for *t* > 1:

$$X_{t}^{i}=X_{t-1}^{i}\left(1+\omega_{t}^{i}\right)+\eta^{i}\left(I_{t-1}-X_{t-1}^{i}\right)\;,$$ (1)

where *η*^*i*^ is defined as the strength of the coupling between the *i*-th stock and *I*_*t*_.

We assume the random sets $\omega_{t}^{i}$ are independent and identically distributed. For results demonstrating the weak effect of this correlation, the reader is referred to S1 Fig.

In order to investigate the results of the model, we initially use a randomwalk to represent *I*_*t*_. Given that *I*_1_ = 1, $I_{t}=\prod_{j=2}^{t}1+\epsilon_{j}$, where for each *j*, *ϵ*_*j*_ follows N(0,0.0001). The choice in this specific statistical noise for the daily return and the daily relative change of the index is originated in the typical mean and standard deviation of the return distribution of several external indices (such as the Baltic Dry Index and the gold price). In practice, the distribution is not normal, however, a more realistic distribution will not affect the model results significantly (see S2 Fig for additional information).

**Fig 1:**
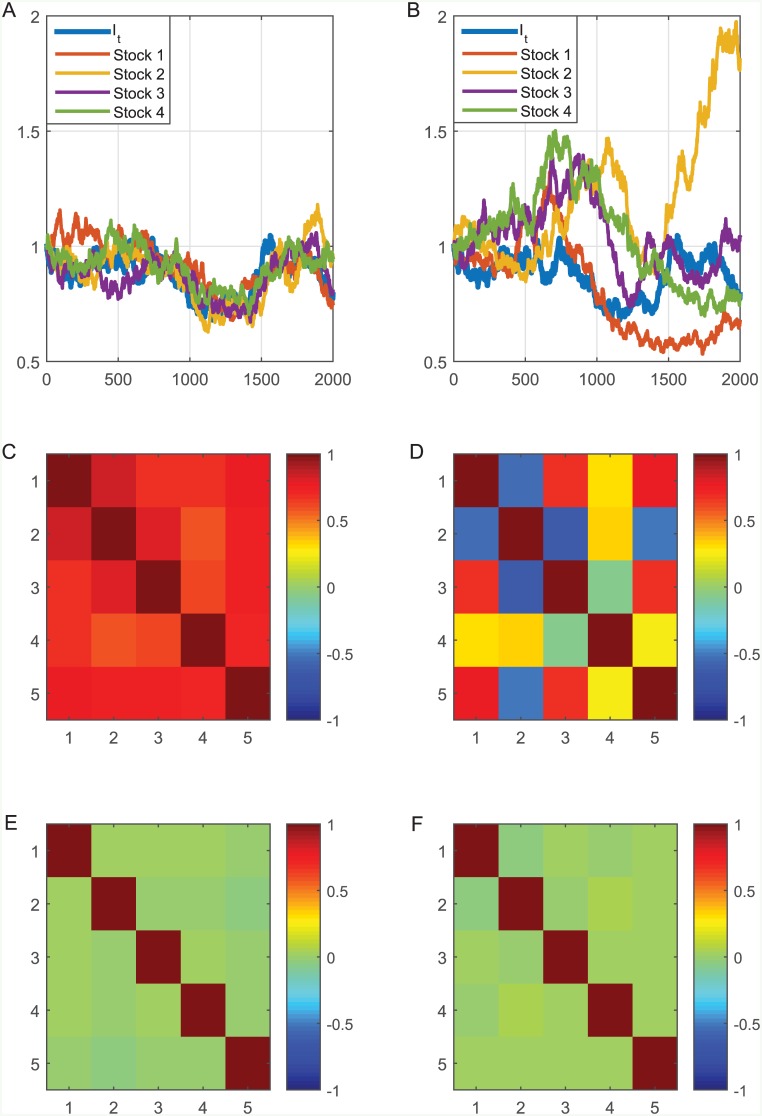
A typical result for the coupling model. A randomwalk was used as *I*_*t*_ (thick blue curve), and following the coupling model, the prices of 4 stocks were constructed and presented for 2000 days and for *η* = 0.01 (A) and *η* = 0 (B). In addition, the correlation structure is presented—the correlation matrices of the price for *η* = 0.01 (C) and *η* = 0 (D) as well as for the return for *η* = 0.01 (E) and *η* = 0 (F) are presented.

A typical result of the model for a random *I*_*t*_ with 4 randomly generated stocks $X_{t}^{i}$ (*i* = 1, 2, 3, 4) is presented in Fig 1 for 2000 days and for 2 values of coupling strength—0.01 and 0 (we considered the coupling *η*^*i*^ to be independent of *i* in this case). It is clearly visible that for *η* = 0, the stocks do not follow the trend of the driving force *I*_*t*_, and in this case the average correlation between the stock prices and *I*_*t*_ is 0.06. The figure shows one typical realization of the model. Averaging over a large number of realizations would produce a price correlation matrix, which is similar to matrices E and F, since the correlation between the different stock prices and *I*_*t*_ would go to zero. This similarity was tested using a *χ*^2^ test, considering 5 degrees of freedom (since there are 5 independent time series) and taking the matrix F as the expected matrix. The averaged price correlation matrix for *η* = 0 and 1000 realizations of the model serves as a good fit to the single realization correlation matrix F with *p* < 0.01.

For *η* = 0.01, the stocks follow the external force trend and a narrower “braid” is obtained. In this case, the average correlation between the stock prices and *I*_*t*_ was 0.86. The correlation between the daily change of the external force and the stocks was also calculated. In both cases it was very low, and practically insignificant: 0.01 for *η* = 0 and -0.01 for *η* = 0.01. In addition, the correlation between the modeled stock prices and a randomwalk was 0 on average, as expected, independent of the value of *η*.

The results for the case *η* = 0.01, depicted in Fig 1, demonstrate that a substantial correlation exists between the stock prices and the index, while the returns are practically uncorrelated. In other words, we obtain a “cohesive” effect of the driving force on the sector stocks in the long run. This behavior is the kind of behavior we would also expect from sectors and their appropriate external driving forces [17–22].

The modeled stock prices produced demonstrate the property of cointegration. We used the Engle-Granger cointegration test [23] to estimate whether the stock prices and *I*_*t*_ meet this property. Depending on *η*, the stock prices analyzed were found as cointegrated with each other, as well as cointegrated with *I*_*t*_ (with *p* < 0.01). For *η* = 0, the stock prices are not cointegrated (*p* > 0.01). Hence, for proper modeling of sectors and their driving forces, in which the stock prices are cointegrated, *η* should be large enough. We point out that cointegration is not a continuous quantitative property such as correlation, and the cointegration property is necessary but not sufficient. In addition, we use the Granger causality test in order to test the relationship between the different time series produced. While other measures of similarity can be also used [24–26], we would mainly use correlation as the continuous quantitative measure to evaluate the similarity between the model results and the real data. We note that the similarity between the external driving forces and the price of a certain stock is more profound than only being strongly correlated. However, within the scope of this work, we reduce the discussion to correlation, cointegration and Granger causality.

**Fig 2:**
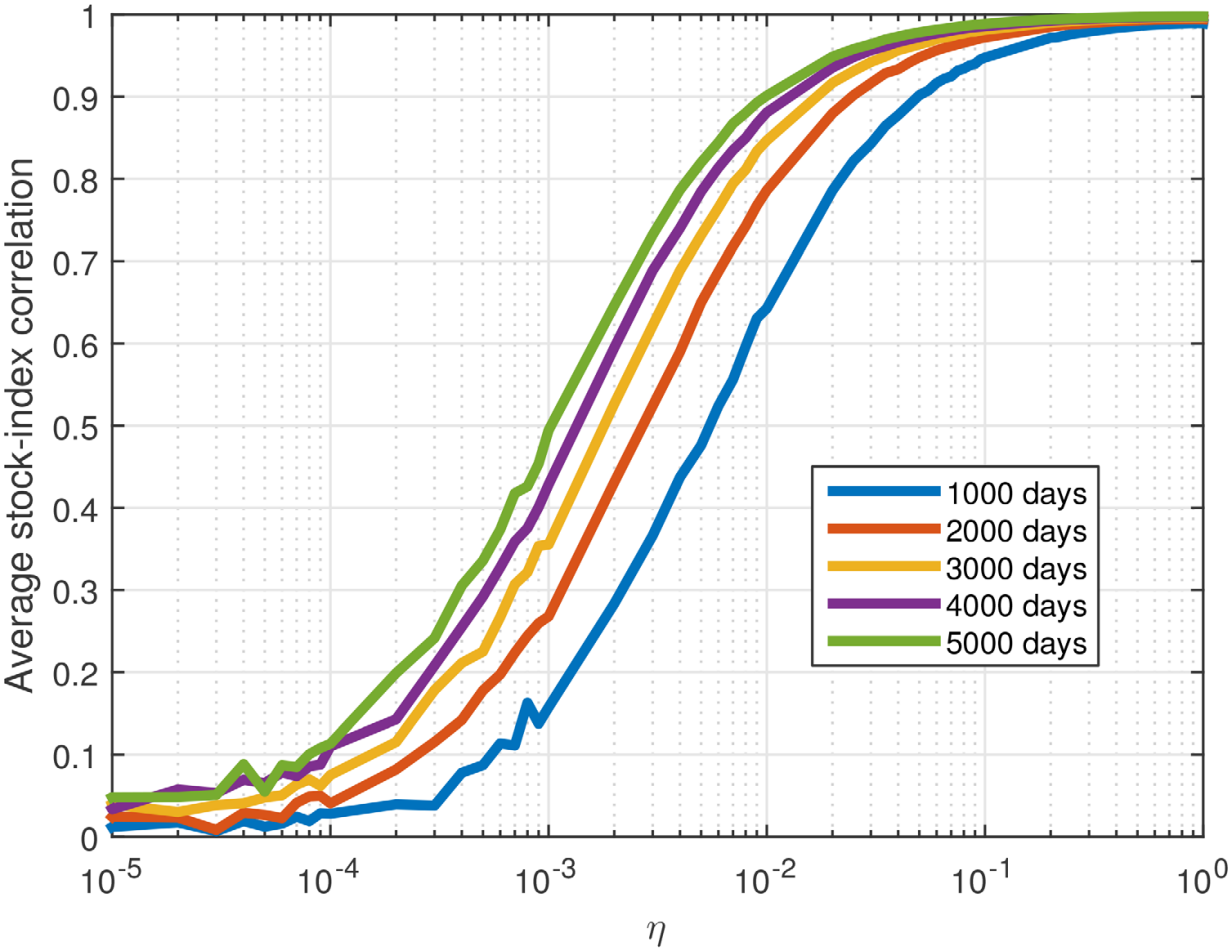
The dependence of the index-stocks correlation on *η*. The average correlation between *I*_*t*_ and arbitrary modeled stock, based on 5000 runs of the model, is presented for several series length (1000, 2000, 3000, 4000 and 5000 days) for *η* running from 0 to 1.

The only parameter in the model is *η*, the coupling intensity. It is responsible for the resulting correlation between the stock prices and the external force. In order to test its effect on this correlation, the dependence of the average correlation between the force and the stock prices was calculated. The results are presented in Fig 2. They demonstrate that as *η* increases, the average correlation increases, with an asymptotic value at 1. The actual values in Fig 2 weakly depend on the variance of *I*_*t*_, while a thorough discussion of the quantitative effects of the variance and other statistical attributes of the external driving forces are left for future work.

## Single factor and mean reverting models in finance

The derivation of the model, described previously, is based on the explained rationale regarding the effect of external driving forces on investors’ decision making, hence on the prices of stocks in various sectors. The resulting model, formulated in Eq (1), is reminiscent of the broad family of single factor models, used extensively in various fields of finance [27–29]. However, these models differ fundamentally from Eq (1), most importantly by modeling the dynamics of stock prices directly. Usually, single factor models are used for modeling return and interest rates and incorporate linear rather than non-linear dynamics.

Another type of model, to which the derived model is very similar, is a mean reversion model type [30]. These models, of which some are also single-factored, are extensively used in various fields such as physics, meteorology and finance. Their financial application is usually for modeling interest rates [28, 31], but also other financial variables characterized by significant positive or negative autocorrelation [32]. A basic discrete mean reverting model inspired by the Ornstein-Uhlenbeck process, a prototype of mean reverting processes, is the following [30]:

$$X_{t}-X_{t-1}=\eta \left(\mu -X_{t-1}\right)+\sigma \left(W_{t}-W_{t-1}\right)\;,$$ (2)

where *μ*, *η* and *σ* are parameters and *W*_*t*_ is a standard Brownian motion (we will refer to Eq (2) as the Ornstein-Uhlenbeck process).

**Fig 3:**
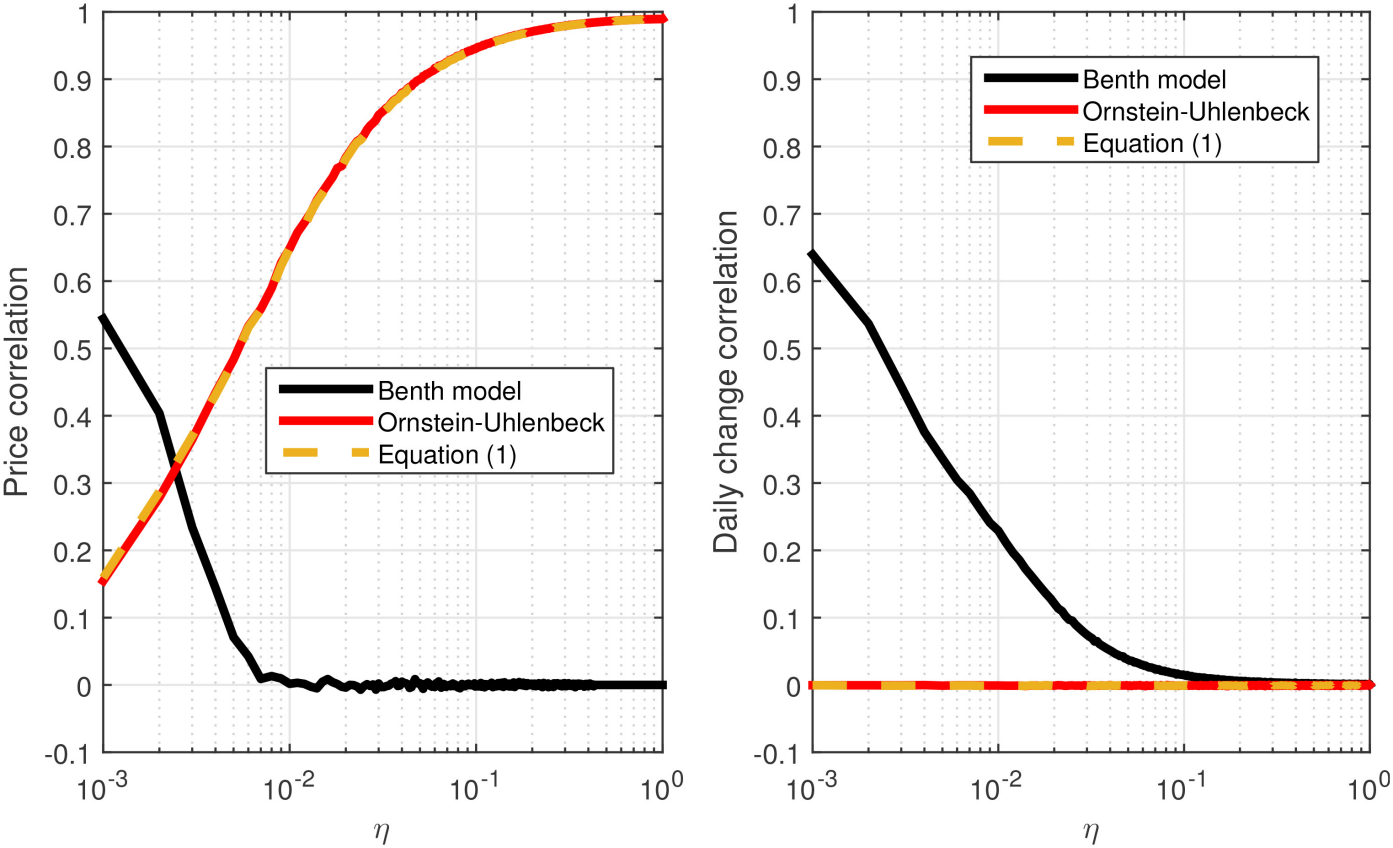
The dependence of the index-stocks price and return correlation on *η* for different models. (Left) The dependence of the index-stocks correlation on *η* for the Benth model (black), the Ornstein-Uhlenbeck process (red) and for the Eq (1) model (dashed yellow curve); (Right) The dependence of the index-stocks return correlation on *η* for the Benth model (black), the Ornstein-Uhlenbeck process (red) and for the Eq (1) model (dashed yellow curve).

The similarity of this process to Eq (1) is clear, however, in this standard version of the process, *μ* is usually considered as constant. If we let *μ* change in time (*I*_*t*_), it becomes almost identical to Eq (1), given that *σ* is taken as constant and similar to the standard deviation of *ω*_*t*_ in Eq (1). We note that in the Ornstein-Uhlenbeck process the random noise is additive and not multiplicative as in Eq (1). However, for practical purposes, when *X* is normalized and close to 1, this difference does not affect the model results, as depicted in Fig 3.

The Benth model [33, 34] is a mean-reverting process and a variation of the Ornstein-Uhlenbeck process, used to model the dynamics of daily air temperatures, but also to model a variety of financial variables [35, 36]. Therefore, we also compared its results to our model. It is defined by the folloping equation:

$$X_{t}-X_{t-1}=I_{t}-I_{t-1}+\eta \left(X_{t-1}-I_{t-1}\right)+\sigma_{t-1}\left(W_{t}-W_{t-1}\right)\;,$$ (3)

where *X*_*t*_ is the daily average temperature, *W*_*t*_ is a standard Brownian motion, *I*_*t*_ is a deterministic function modeling the trend and seasonality of the average temperature, while *σ*_*t*_ is the daily volatility of temperature variations.

We compared between Eqs (1–3), given the same value of *σ* (or standard deviation of *ω*_*t*_). 10000 reference time series, *I*_*t*_, of 1000 time steps (or days) were considered for different values of *η*. The reference time series were defined as done in the previous section (*I*_1_ = 1; $I_{t}=\prod_{j=2}^{t}1+\epsilon_{j}$, where for each *j*, *ϵ*_*j*_ follows N(0,0.0001)). We used the 3 models to construct a time series *X*_*t*_. We calculated the correlation between *X*_*t*_ and *I*_*t*_ and between the daily relative change of each of these series. For each value of *η* we considered the average value of these correlations (within the 10000 *I*_*t*_ series), so we could compare the results depicted in Figs 1 and 2 between the different models. The results of the comparison are presented in Fig 3.

As demonstrated by the results in Fig 3, the results for Eq (1) and for the standard version of the Ornstein-Uhlenbeck process (for non constant *μ*) are almost identical. The Benth model is naturally inappropriate for modeling the effect of driving external forces on the price in the way described in the previous section.

It is possible to produce a smoothed time series for each *I*_*t*_ using LOWESS method [37]. The similarity of the results for *I*_*t*_ and for the smoothed time series, $\tilde{I_{t}}$, is found to be very high (see S3 Fig) and preserves the fundamental property of high stock-index price correlation and 0 correlation in the return. Using a smoothed *I*_*t*_ is a more realistic assumption than considering the value itself, since in practice a stock price can significantly diverge from the external force value. However, proper normalization eliminates the need for considering a smoothed approximate time series for the external driving force. In the next sections, the financial data analyzed will be normalized so that the average value of the stock price equals that of the index. This normalization is necessary, as otherwise the expression $\left(I_{t-1}-X_{t-1}^{i}\right)$ in Eq (1) is essentially meaningless.

## The shipping market as a test case

Following the model derivation, a statistical characterization of market sectors is essential in order to validate it. We will try to capture the unique statistical properties of stocks that belong to a certain sector and traded in stock markets. Such stocks can belong not only to the same sector but also to the same market (for example, if traded in the same stock exchange). Therefore, each stock can be characterized according to several categories of belonging, most notably its stock market index and the driving force dominating its sector.

In order to perform this characterization, we first address the shipping sector, which functions as a test case for a sector dominated by an external force. By characterizing this sector specifically, we will be able to derive general conclusions regarding the nature of sectors and compare these conclusions with the model results.

The shipping stock market is a unique sector in the financial market, in which stock prices are ultimately regulated by external parameters. Most notably, these parameters include fuel prices, raw material and infrastructure demand as well as various commodity prices [19, 38]. Because the international shipping industry facilitates 90% of world trade and is a key factor in global economic development, it is a major factor in economic and financial theory [39–41]. The shipping industry is tightly linked to the world economy and to the international trade business cycle. Therefore, it enjoyed a long prosperous period with growing trade at the international level until the financial crisis in 2008 [41–44]. The shipping industry is also dynamic and volatile. The volatility in shipping markets is significantly higher (0.79%) than the average volatility in commodity markets (0.5%) and equity markets (e.g., S&P500 0.2%) [44]. This extremely high risk is not only due to volatility in global economic cycles, but is also highly influenced by intrinsic characteristics of the shipping industry itself [19]. Naturally, the shipping market stocks are linked both to the stock market, represented by the stock market index, and to external driving forces.

The *Baltic Dry Index* (BDI) [44] is an index issued daily, which provides an assessment of the price of transporting dry cargo (such as grains, coal, ore and cement) by sea, representing the physical market. Its value is measured in US dollars (though arbitrary, in fact) and determined by a panel of international shipbrokers. As such, important information is contained in its value, substantially affecting the shipping market.

**Fig 4:**
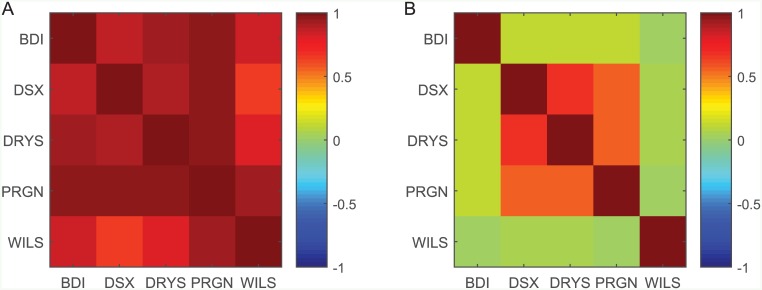
The correlation structure in the shipping market. The correlation in the price (A) and in the daily change (B) between the Baltic dry index and stocks of 3 shipping companies traded in New York stock exchange (NYSE)—DSX, DRYS and PRGN—and one in Oslo stock exchange—WILS—for the period 2007–2014 (see S1 Table).

In order to analyze the relationship between the BDI and different shipping market stocks, the correlation between them was calculated. We used 4 different stocks—DSX, DRYS, PRGN and WILS, 4 leading shipping companies focusing on dry bulk market, and calculated the correlations for the period 2007–2014. The results are presented in Fig 4.

**Fig 5:**
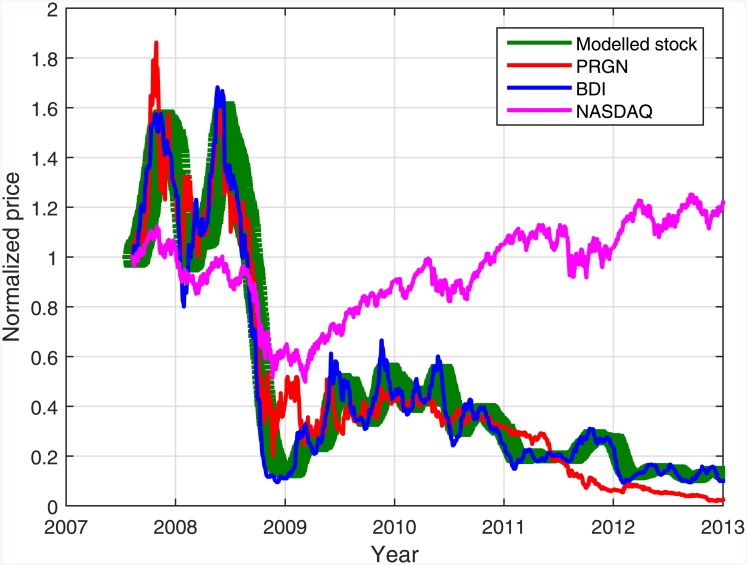
The simulated PRGN stock price. The BDI is displayed (blue) along with the real PRGN stock price (red) and the averaged price of 1000 model realizations using *η* = 0.062. The error bars signify ±1*σ* (green). Also presented is the NASDAQ Composite index historical value (magenta).

The correlation between the BDI and the price of the shipping companies examined is very high, meaning that indeed, the BDI reflects relatively well the state of the shipping industry. However, there is only a weak positive correlation between the daily change of the BDI and the daily return of the stocks. It should be noted, in addition, that the correlation between these stocks and the BDI is much higher (0.8–0.9) than the positive correlation that exists between stocks and the stock market index (0.2–0.4) [20, 45–49]. Therefore, the high correlation found cannot be explained by the overall market behavior, although it affects both the stocks of shipping companies and even the BDI, but only weakly. This is also illustrated in Fig 5, in which the historical PRGN stock price is presented along with the BDI and the NASDAQ Composite index to which the PRGN stock belongs. The positive correlation between the stock price and the stock market index (0.2) is much lower than the correlation between the stock price and the BDI (0.96). However, when the daily relative change is compared, we obtain a correlation of 0.5 between the daily relative change of the PRGN stock price and the NASDAQ Composite index, and 0.1 between the PRGN stock price and the BDI daily change.

The correlation matrices depicted in Fig 4 are similar in structure and values to the model results presented in Fig 1. The similarity was also tested using a *χ*^2^ test, considering 5 degrees of freedom, taking the correlation matrices presented in Fig 4 as the expected matrices. It was found that both the stock price correlation matrix and the daily return correlation matrix in Fig 1 serve as a good fit to the real correlation matrices with *p* < 0.01. This demonstrates that the model can represent the actual financial data regarding the correlation structure in market sectors, particularly in the shipping sector.

In addition, the stock prices analyzed were found as cointegrated with each other, as well as cointegrated with the BDI value (with *p* < 0.01) and all the stocks are found to Granger cause all others and the BDI (and vice versa) with *p* < 0.01. These properties are most probably originated by the substantial effect of the BDI on the stocks. Their return, however, as implied from Fig 4, is not cointegrated with the daily changes of the BDI.

Together with the previous results, a clearer picture of the dynamics of the shipping market emerges. On the daily time scale, the BDI is not a strong effector on the shipping market stocks. The daily returns of the stocks are governed by the fluctuative nature of stock market trade, by news flow and by the patterns of the investors’ behavior [20, 50, 51]. However, on a longer time scale, such as the monthly or annual time scales, the trend of the stocks is close to the trend of the BDI, which represents the state of the physical shipping market.

The model results and the analyzed data demonstrate that the model produces results that agree with the statistical properties of the market sectors, as presented for the shipping market. In particular, the modeled stocks present high correlation (as a function of *η*) with the value of the external driving force, while their daily return is independent of the daily change of the force. Consequently, it is observed that a mechanism such as the coupling term in the model can explain the high correlation between the external force and the stock prices on a long time scale, without changing the uncorrelated behavior on shorter time scales.

We note that using a normal distribution for the daily change is not based on real data. The real return distribution approximately follows a Lévy distribution [20]. However, this difference is insignificant in the context of the behavior analyzed (see S2 Fig for additional information). The realistic return distribution does not contradict the model results, yet it is simply unnecessary for demonstrating the presented effect of the external forces on stock prices. Therefore, for simplicity, a normal distribution is used.

Following these results it is possible to estimate the value of *η* for specific driving forces and stocks, and particularly, for the shipping market. The average correlation between the BDI and the analyzed shipping market stocks was 0.89, which approximately corresponds to *η* = 0.025, according to the model results. If the BDI is taken as *I*_*t*_ we can produce simulated prices of “shipping” stocks. The model was used to create a simulated time series for the PRGN stock, using the appropriate number of days (1772) and the appropriate value of *η* corresponding to the correlation between the PRGN stock price and the BDI (*η* = 0.062). Fig 5 presents this calculation.

The results demonstrate that the simulated stock prices highly resemble the original stock price and their average correlation with the BDI is almost identical (0.955) to the correlation of the original stock (0.956). The correlation between the PRGN stock price and the simulated stock prices was 0.97 on average. As expected, the daily return of these two stocks displays an insignificant correlation (0.04). The appropriate value taken for *η* (0.062 or 6.2%) lies in a sensible domain of values [52, 53]. Furthermore, the difference between the various realizations is very small. The modeled stock price time series was also found to Granger cause the BDI time series with *p* < 0.01, as expected.

The results presented so far indicate that the coupling model devised captures the statistical characteristics of market sectors and can be applied for the shipping market. By properly adjusting the value of *η*, an artificial stock price time series is created, such that its quantitative behavior is similar to real stock prices within the shipping market. This confirms the underlying assumptions of the model, and the mechanism by which the external driving force effects the sector stock prices.

## Analysis of the gold mining and the food production markets

In order to further validate the model, the same analysis previously applied to the shipping market was also applied to the gold mining and food production markets. For the gold mining market the gold price was taken as the external force *I*_*t*_, and the stock prices of 4 leading gold mining companies were considered—Goldcorp Inc. (GG), Barrick Gold Corporation (ABX), Silver Wheaton Corporation (SLW) and Franco-Nevada Corporation (FNV). For the food production market we used the wheat price as the external force, and the stock prices of 4 grains and food manufacturing companies were considered—Archer Daniels Midland Company (ADM), Bunge Limited (BG), Nestlé S.A. (NSRGY) and Ingredion Inc. (INGR). For more details, refer to S1 Table.

**Fig 6:**
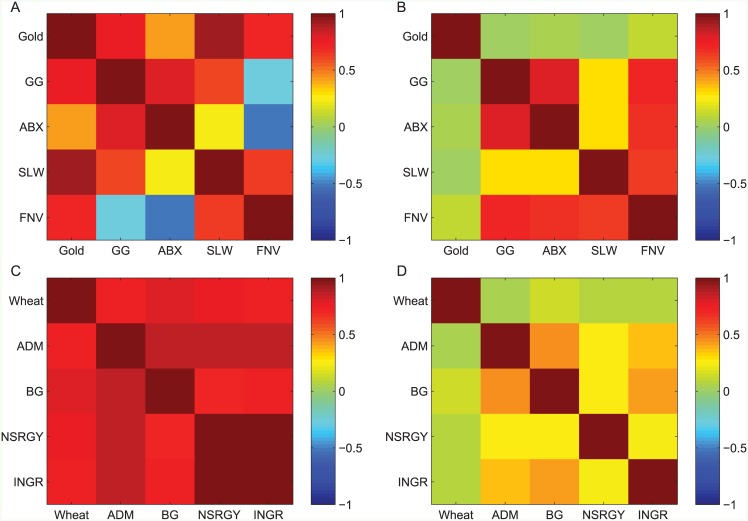
The correlation structure in the gold mining and food production markets. The correlation in the price (A) and in the daily change (B) between the gold price and stocks of 4 leading gold mining companies traded in NYSE and Toronto stock exchange for the period 2007–2014; The correlation in the price (C) and in the daily change (D) between the wheat price and stocks of 4 leading food companies traded in NYSE and over the counter for the period 1983–2014.

The correlations between the different stocks and the external forces were calculated. This calculation was done both between the stock prices and the external force values and between the stock returns and the external force relative daily change time series, as done for the shipping market stocks. The results are presented in Fig 6.

The correlation between the gold price and the price of the gold mining companies examined, as well as for the wheat price and food companies, is very high (except for ABX). As implied by the results for the shipping market, the results demonstrate that the gold mining and food production markets can be regarded as examples for sectors with an external driving force (compare with Figs 1 and 4). There is only a very weak positive correlation between the daily change of the external force and the daily return of the stocks, as found for the shipping market. In addition, all the stock prices (apart from FNV in the gold mining sector) within each of the sectors are found to be cointegrated and Granger cause each other (with *p* < 0.01).

**Fig 7:**
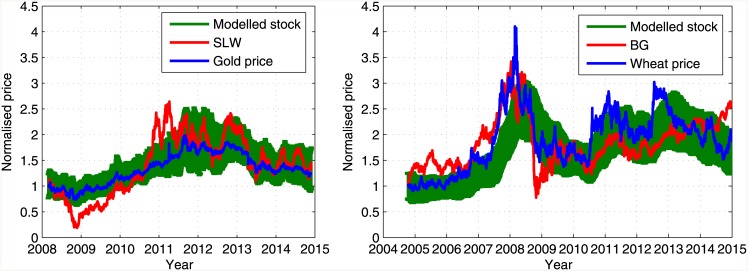
Simulated gold mining and food production stock prices. (Left) The gold price is displayed (blue) along with the real SLW stock price (red) and the averaged price of 1000 model realizations using *η* = 0.02 (green); (Right) The wheat price is displayed (blue) along with the real BG stock price (red) and the averaged price of 1000 model realizations using *η* = 0.006 (green). The error bars signify ±1*σ*.

The model was applied to the gold mining market as previously done for the PRGN stock taking the SLW stock for the gold mining sector and the BG stock for the food production sector. The results are presented in Fig 7, capturing a similar behavior to the behavior depicted in Fig 5.

Due to the lower correlation between the stock prices and the driving forces in the gold mining and food production sectors, when compared to the shipping sector, the modeled stock prices obtained are reminiscent of the original stock price. This indicates that the prices of these stocks might be driven by additional driving forces, in particular the stock market index. For example, the correlation between the BG stock price to the S&P500 index to which it belongs, is 0.72, which is almost as high as its correlation to the wheat price. Therefore, in practice, in order to provide a proper model for the stock price, an additional coupling parameter is needed to an additional external driving force. This expansion of the model is left for future work. We note, however, that for most of the stocks, the correlation between their price and the stock market index is about 0.2 to 0.4 [20].

## Discussion

We described the statistical properties of market sectors with an external driving force. These sectors are most notably characterized by a long time scale dependence on the driving force, meaning that the trend of the force strongly affects the sector stock prices. However, the short term behavior of the stocks is weakly affected by this force. In particular, the daily return of the sector stocks is effectively independent of the daily change of the external force. The daily return is therefore a result of the market behavior [20, 50]. In addition, the stock prices are found to be statistically cointegrated and to Granger cause each other as well. These properties were demonstrated by analyzing the shipping sector, the gold mining sector and the food production sector.

This characteristic behavior motivated us to devise a mean reverting model based on the coupling between sector stock prices and a driving force for the sector stock price dynamics. The model was found successful by reproducing all the described properties. By adjusting one free parameter—the coupling intensity between the external force and the stock prices—we were also able to artificially reproduce the actual stock prices, preserving the statistical properties demonstrated. We note, however, that other statistical properties related to short term characteristics of financial data, such as intraday behaviors, the return distribution and the slow decay of the autocorrelation with the lag, are irrelevant within the scope of this model. At this stage, we only capture the behavior of stock prices within sectors, which are affected by external forces and unaffected by these statistical properties.

The findings of this work, together with the study of short term herd behavior phenomena in the stock market [20], demonstrate that the full description of the dynamics of stocks, can be done by integrating multiple time scales and multiple dominating forces or indices [48]. Notably, we found that along with short time scale coupling between stock prices to the stock market index [18, 20, 48], there exists a longer time scale coupling to a sector specific external force.

The results imply that some market sectors are essentially governed by driving forces, which are sometimes independent of the market general behavior. For example, the BDI, the gold price and the wheat price are independent of the S&P500 index, which is considered a bellwether for the U.S. economy. Therefore, the understanding of such sectors might contribute to the diversification of risk in the financial market, notably under market stress. Empirical observations demonstrate that in times of stress, correlations within the financial markets increase [54], so that on a long time scale, sectors governed by external driving forces, might reduce the importance of such effects [11–13].

It follows that each stock has several categories of belonging, corresponding most notably with its stock market index and an external driving force dominating its sector. For example, when analyzing the shipping market we considered the stock WILS, which is traded in the Oslo stock exchange. WILS demonstrated a very low correlation between its daily return and the daily return of the other shipping stocks considered, which are traded in the NYSE. However, on a longer time scale, the prices of WILS and the other stocks were highly correlated, due to the effect of the BDI. In addition, the stock index might also have an effect on stock prices on a longer time scale which is smaller than the effect of the BDI or the external driving forces in general. Such an effect might be associated with the discrepancy depicted in Figs 5 and 7 between the modeled stock price and the real stock price. Introducing an additional coupling parameter between the modeled stock price and the stock market index might improve the model accuracy. Following this, a more intricate measure of similarity between the stock price, the index and the external driving force should be introduced, as correlation will be insufficient as such measure in this case.

Following Eq (1), and by omitting the statistical noise, the parameter *η* can be mathematically interpreted as the reciprocal of a characteristic time scale. For example, taking a time interval of one day, *η* = 0.01 can be associated with a characteristic time of 100 days. This time characterizes the process of the stock price convergence into the driving force value. The statistical noise plays an important role and therefore this interpretation is not exact. However, it introduces a characteristic time scale to the dynamics described by the model. The values of *η* associated with the sectors presented previously, stand for characteristic time scales ranging approximately from 25 to 200 days, and confirm the notion of long term effect of the external driving forces.

More research should be carried out to establish the above: The integration of the model with the herd behavior model for the shorter time scale [20] to provide a model consistent with market phenomena on a variety of time scales; expansion of the model to include an approximate smoothed time series for the external force to provide a more realistic driving effect on the stock prices; expansion of the model to include additional important statistical attributes of the driving forces; further validation of the model by considering additional economies and sectors; considering additional measures of similarity between stock prices and driving forces; relating the results to risk assessment, specifically in times of financial stress or crisis.

## Supporting Information

S1 Fig The model results with and without stock-stock return correlation. A randomwalk was used as *I*_*t*_ (thick blue curve), and following the presented coupling model, the prices of 4 stocks were constructed and presented for 1000 days and for *η* = 0.05. It was taken without any correlation between the daily returns of the different stocks (A) and given the actual correlations for the shipping stocks analyzed (see Fig 4 in the paper) (B). In addition, the correlation structure is presented—the correlation matrices of the price without (C) and with daily return correlation (D), as well as for the daily return without (E) and with daily return correlation (F).
(EPS)

S2 Fig Comparison between normal and Lévy distributed returns. (A) The dependence of the index-stocks correlation on *η* for normal distributed returns (blue) and Lévy distributed returns (green); Also presented are the correlation matrices of the price for *η* = 0.01 (B) and the return (C) for the Lévy distributed returns. Compare these results to Fig 1 in the paper. The parameters used for the Lévy distributed return were taken from the analysis of the Baltic dry index daily change (for the external force) and from typical values for the daily return of stock traded in the stock market (for the stocks).
(EPS)

S3 FigThe dependence of the index-stocks price and return correlation on *η* for different models.(A) One realization of *I*_*t*_ and $\tilde{I_{t}}$ time series, smoothed using the LOWESS method; (B) The dependence of the index-stocks correlation on *η* for the Benth model (black), the Ornstein-Uhlenbeck process (red), Eq (1) model (dashed yellow curve) and for Eq (1) model calculated using $\tilde{I_{t}}$ (green); (C) The dependence of the index-stocks return correlation on *η* for the Benth model (black), the Ornstein-Uhlenbeck process (red), Eq (1) model (dashed yellow curve) and for Eq (1) model calculated using $\tilde{I_{t}}$ (green).(EPS)

S1 Table Summary of all data sets used in the empirical analysis for the model validation. In order to validate the model, we use empirical data and compare to model results to the characteristics of different sectors in the market. The data used is for the dry shipping market, the gold mining sector and the food production sector and based on various databases [55–57]. The following table summarizes all the data sets used in this analysis and their sources.
(PDF)
