# Supplementary material for: Analyzing the Long Term Cohesive Effect of Sector Specific Driving Forces
Source: PLoS One. 2016 Mar 31;11(3):e0152487. doi: 10.1371/journal.pone.0152487 (PMC4816528; doi:10.1371/journal.pone.0152487)
Supplement: S1 Table — In order to validate the model, we use empirical data and compare to model results to the characteristics of different sectors in the market. The data used is for the dry shipping market, the gold mining sector and the food production sector and based on various databases [55–57]. The following table summarizes all the data sets used in this analysis and their sources. (PDF) [file pone.0152487.s004.pdf]

Data series	*Ticker / Abbreviation*	*External force / asset*	*Tradable (Yes/No); Stock exchange*	*Source*
Baltic Dry Index	BDI	External force	No	Clarkson's SIN database [53]
Diana Shipping	DSX	Asset	Yes; NYSE	Yahoo! Finance [54]
DryShips	DRYS	Asset	Yes; NYSE	Yahoo! Finance [54]
Paragon Shipping	PRGN	Asset	Yes; NYSE	Yahoo! Finance [54]
Wilson Ship Management	WILS	Asset	Yes; Oslo Børs	Yahoo! Finance [54]
Gold price	-	External force	No	Yahoo! Finance [54]
Goldcorp	GG	Asset	Yes; NYSE	Yahoo! Finance [54]
Barrick Gold	ABX	Asset	Yes; Toronto Stock exchange	Yahoo! Finance [54]
Silver Wheaton	SLW	Asset	Yes; NYSE	Yahoo! Finance [54]
Franco-Nevada	FNV	Asset	Yes; NYSE	Yahoo! Finance [54]
Wheat price	-	External force	No	Nasdaq database [55]
Archer Daniels Midland Company	ADM	Asset	Yes; NYSE	Yahoo! Finance [54]
Bunge	BG	Asset	Yes; NYSE	Yahoo! Finance [54]
Nestlé S.A.	NSRGY	Asset	Yes; Swiss Exchange	Yahoo! Finance [54]
Ingredion	INGR	Asset	Yes; NYSE	Yahoo! Finance [54]
